# The Distribution of Trace Metals in Roadside Agricultural Soils, Thailand

**DOI:** 10.3390/ijerph16050714

**Published:** 2019-02-27

**Authors:** Nattanan Krailertrattanachai, Daojarus Ketrot, Worachart Wisawapipat

**Affiliations:** Department of Soil Science, Faculty of Agriculture, Kasetsart University, Bangkok 10900, Thailand; nattanan.k@ku.th (N.K.); worachart.w@ku.th (W.W.)

**Keywords:** roadside agricultural soils, trace metals, distance from the road, contamination factor

## Abstract

Vehicle emissions have been known to cause trace metal contamination in soils. The extent of such contaminations in soils, and of the effects of traffic density and distance from highways on the concentration of trace metals in roadside agricultural soils is largely unknown. This study examined the total concentrations of common trace metals (Cd, Co, Cr, Cu, Ni, Pb, V, and Zn) in roadside agricultural soils from Thailand with diverse traffic densities (approximately 30–200 million vehicles/kilometer/year), roadside distances (0, 10, 20, 50, and 100 m from the road edge), and crops (rice, maize, and sugarcane). Cadmium, Cu, Pb, and Zn concentrations significantly decreased with increasing distance away from the roads (*p* < 0.05). However, the concentrations of these metals were not correlated with traffic density, probably due to extensive road maintenance and expansion. The contamination factor demonstrated that the road edge soils were moderately- to highly-polluted with Cd, Cu, Pb, and Zn. The safest distance to minimize metal pollution for agricultural production is proposed to be greater than 10 m away from the road edge.

## 1. Introduction

Traffic is a primary source of trace metal pollution in roadside soils, and plays a pivotal role in the biogeochemical cycling of trace metals, and in turn substantially affects human health [1,2,3]. Trace metal contamination can be found in road dust and roadside soils. Road dust commonly contains high concentrations of Pb, Zn, Cr, Cu, Ni, As, Cd, and Hg [4,5,6,7], whereas roadside soils are frequently contaminated with Pb, Zn, Cd, Ni, Cr, Cu, and As [8,9,10,11]. Fuel combustion, and brake pad and tire abrasion have been documented as the primary contributors of metals in roadside soils. Fuel and lubricant oil combustion can considerably emit atmospheric particulates that are abundant in trace metals such as Pb, Cd, Cr, Cu, Ni, V, As, and Hg [12,13]. Tire and engine oil production utilize Zn as an additive; tire attrition and engine oil leakage can cause Zn contamination in roadside soils [7,14,15]. Furthermore, tire corrosion can release a number of trace metals, such as Cd, Co, Cr, Cu, Hg, Mn, Mo, W, Ni, and Pb [7,16,17]. Brake abrasion releases Zn, Fe, Ti, Cu, Ba, Mo, and Zr to the soils [16,17].

The distribution patterns of trace metals in soils have been found to be associated with the distance from the road. A number of studies have demonstrated that trace metal concentrations typically decrease with increasing distance from the road [5,8,9,18]. Traffic activities accelerate trace metal accumulation in roadside soils, however, the effect of vehicles on soil contamination is limited at 50–100 m of the road edge for non-agricultural roadside soils and at 3–10 m for roadside agricultural soils [19,20,21,22]. Traffic density and road age also impact trace metal concentrations in soils. High traffic densities and old roads contribute greater metal concentrations to roadside soils than low traffic densities and new roads [18,23]. Roadside soils along medium-aged roads (10–15 years old) and new roads (2–5 years old) of western Melbourne in Australia accumulated high concentrations of Mn, Sb, and Pd, while soils along the old roads (>30 years old) were highly polluted with Pb, Zn, and Cr [23]. Lead concentrations in roadside soils were significantly (*p* < 0.05) correlated with vehicle volume [8], as Pb was previously added to increase the octane value of fuel [21].

Thailand is an agricultural country, and roadside soils are often being used for economic crop production, such as rice, maize, and sugarcane. However, little research has been done in relation to trace metal concentrations in roadside agricultural soils. Therefore, the objectives of this study were to evaluate the concentrations of trace metals in roadside agricultural soils and to examine the effects of vehicle density and the distance from the road on the concentrations of trace metals contamination in roadside agricultural soils. The results from this research could provide a suitable distance from the road for crop production to help reduce the pollution risk of trace metals from traffic.

## 2. Materials and Methods

### 2.1. Study Area

**Figure 1:**
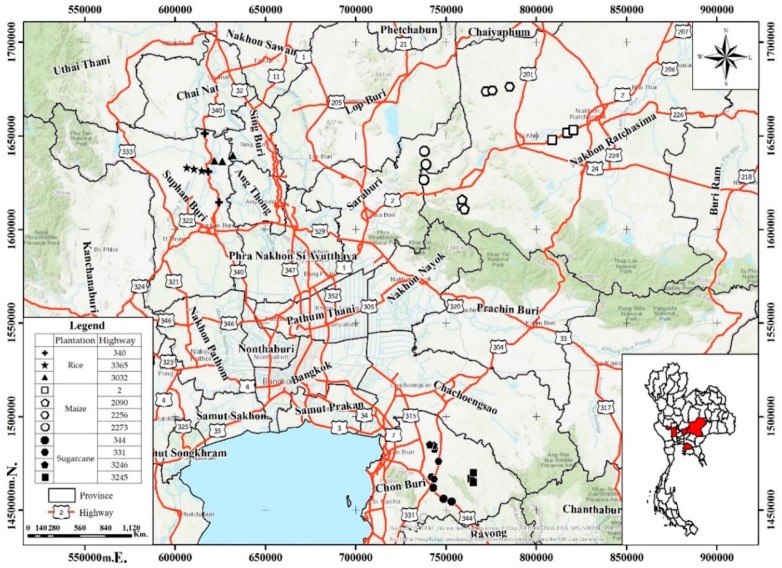
Sampling sites of roadside agricultural soils from central, northeastern and eastern Thailand.

Samples of roadside agricultural soils in this study were obtained in 2016, and the sampling sites are shown in Figure 1. The roadside sites are used for major economic crop production consisting of rice, maize, and sugarcane. Rice growing soils were sampled from Suphan Buri and Sing Buri provinces located in central Thailand, maize growing soils from Saraburi and Nakhon Ratchasima provinces located in north-eastern Thailand, and sugarcane growing soils from Chonburi province located in eastern Thailand. The traffic density for the sampling sites ranged from, approximately, 30–200 million vehicles/kilometer/year (Table S1) according to the criteria of the Bureau of Highway Safety [24,25].

### 2.2. Soil Sampling and Preparation

Three transects were sampled next to each highway, and for each transect soil samples were obtained at five distances—0, 10, 20, 50, and 100 m—from the road edge. The soil samples were taken from the surface to the base of the Ap horizon, except for the road edge sample (0 m distance) that was taken from 0–10 cm. The road edge samples were considered as representative of the trace metal concentrations released from motor vehicles. A total of 165 soil samples were collected and stored in plastic bags for laboratory analysis. Soil samples were air-dried, gently disaggregated, and sieved through a 2 mm sieve to remove gravel, plant debris, and plant litter. The sieved soil samples were used for particle size distribution using the pipette method and general chemical analysis, except for the determination of organic carbon and total metal concentrations, where the sieved soil was finely ground to <0.5 mm. The methods used for the soil analyses are described in Soil Survey Staff [26]. Soil pH was measured in 1:1 (soil: water suspensions) and electrical conductivity (ECe) was measured in a saturation extract. Cation exchange capacity (CEC) was determined by 1M NH_4_OAc (pH 7.0). Organic carbon content was determined using the dichromate oxidation procedure, then multiplied by the Van Bemmelen factor of 1.724 to calculate organic matter [26].

### 2.3. Total Trace Metal Concentrations

Total trace metal concentrations of soil samples were determined by a wet digestion procedure using a mixture of HNO_3_:HClO_4_ (5:2). One gram of each sample (0.5 mm sieve) was weighed into a digestion tube, then 10 mL of the mixed acids was added to the digestion tube, and the sample was open digested at 150 °C for 3 h, or until a clear solution was obtained. The final volume was made up to 25 mL. The clear solution was preserved, and subsequently diluted before the elemental analysis. Total concentrations of Cd, Co, Cr, Cu, Ni, Pb, V, and Zn were determined by inductively coupled plasma-optical emission spectrometer (ICP-OES) (Optima 8300, PerkinElmer, Waltham, MA, USA) using an axial view of the plasma. Standard solution (PerkinElmer Number N9300281) for the ICP was used for preparing calibration curves and used as the internal standard. Metal concentrations in the digests were checked against stream sediment reference material (STSD-3) (Canadian Certified Reference Materials Project) for quality assurance. The deviation between standard replicates (*n* = 3) for the metal concentrations was within 0.98–3.97% RSD (mean = 2.02%).

### 2.4. Contamination Factor

The contamination factor (CF) was used to assess the enrichment of trace metals in the soils and to measure the pollution levels [9,27]. The CF was calculated using Equation (1), where CF_i_ is the contamination factor for a trace metal (Cd, Co, Cr, Cu, Ni, Pb, V, and Zn), C_i_ is the concentration of the trace metal in the sample, and S_i_ is the background concentration of the trace metal in the soil sample at a distance of 100 m from the road edge [19,20,21,22]. The CF was classified into four levels of pollution: CF < 1 represents no or minimal pollution; 1 ≤ CF < 3 indicates moderate pollution; 3 ≤ CF ≤ 6 indicates considerable pollution; and CF > 6 indicates very high pollution [9,27]:

CF_i_ = C_i_/S_i_ (1)

### 2.5. Statistical Analysis

The trace metal concentrations were analyzed using a one-way analysis of variance (ANOVA), and the mean values were compared among sampling distances for each highway using Duncan’s Multiple Range Test (DMRT) at *p* < 0.05. A Pearson correlation was also performed at *p* < 0.05 and *p* < 0.01. A principal component analysis (PCA) was performed to demonstrate the association of metal concentrations with relevant soil properties and related factors.

## 3. Results and Discussion

### 3.1. General Soil Characteristics

The studied soils developed over time on alluvium, sandstone, limestone, and granite. These soils were moderately acidic to moderately alkaline (pH 5.69–7.93), and non-saline (ECe 0.2–2.4 dS m^−1^). The organic matter (OM) content varied from low to moderately high (7–61 g kg^−1^), while the cation exchange capacity ranged from low to high (3.5–28.0 cmol_c_ kg^−1^) (Table S2). Most of the road edge soils (at a distance of 0 m) from all plant types were more coarse-textured, had a higher pH and OM content, and had lower CEC values than the other soils (soil at the distances of 10, 20, 50 and 100 m from the road edge). This was due to the effect of road construction materials, such as sand, rock, and cement, contributing to large amounts of coarse soil particles, and therefore a high sand content, high pH, and low CEC. The high OM concentration in the road edge soils was due to the presence of local grass on road edges.

### 3.2. Total Concentrations of Trace Metals in Roadside Agricultural Soils

**Figure 2:**
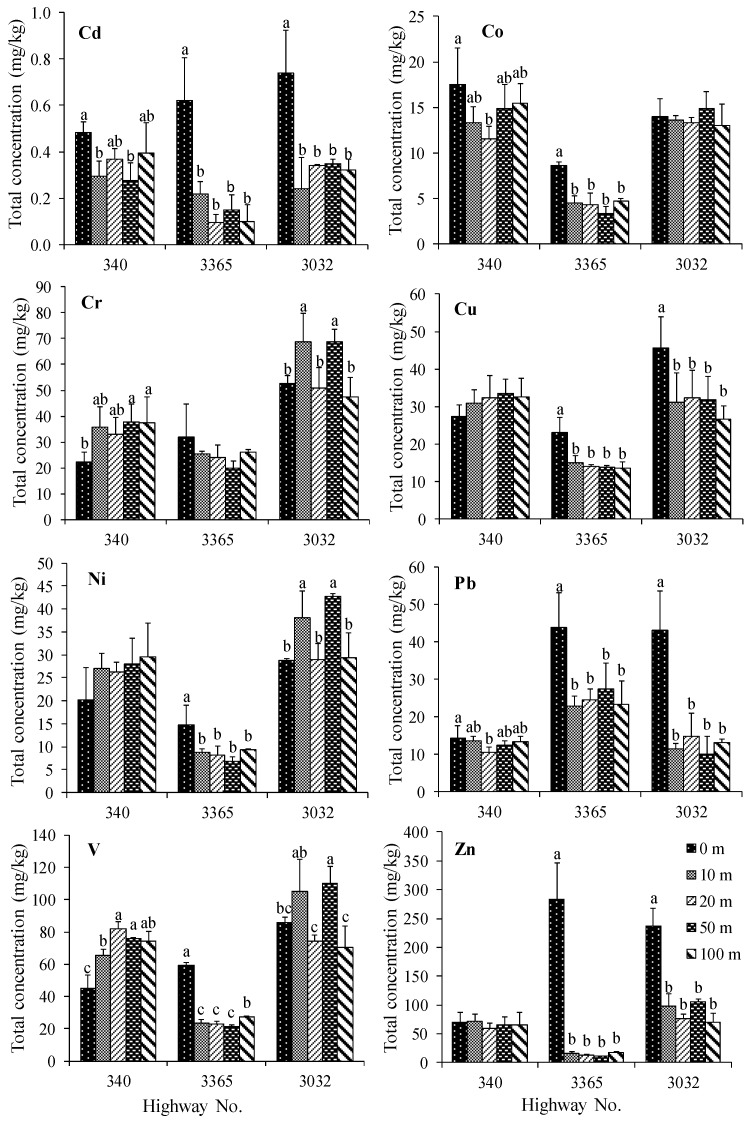
Mean total concentrations of Cd, Co, Cr, Cu, Ni, Pb, V, and Zn (mg kg^−1^) in rice growing soils at the different distances from the road edge. The columns with different alphabets indicate significant differences and those without alphabets indicate insignificant differences in the soil concentrations (*p* < 0.05), and error bars indicate ±SD.

**Figure 3:**
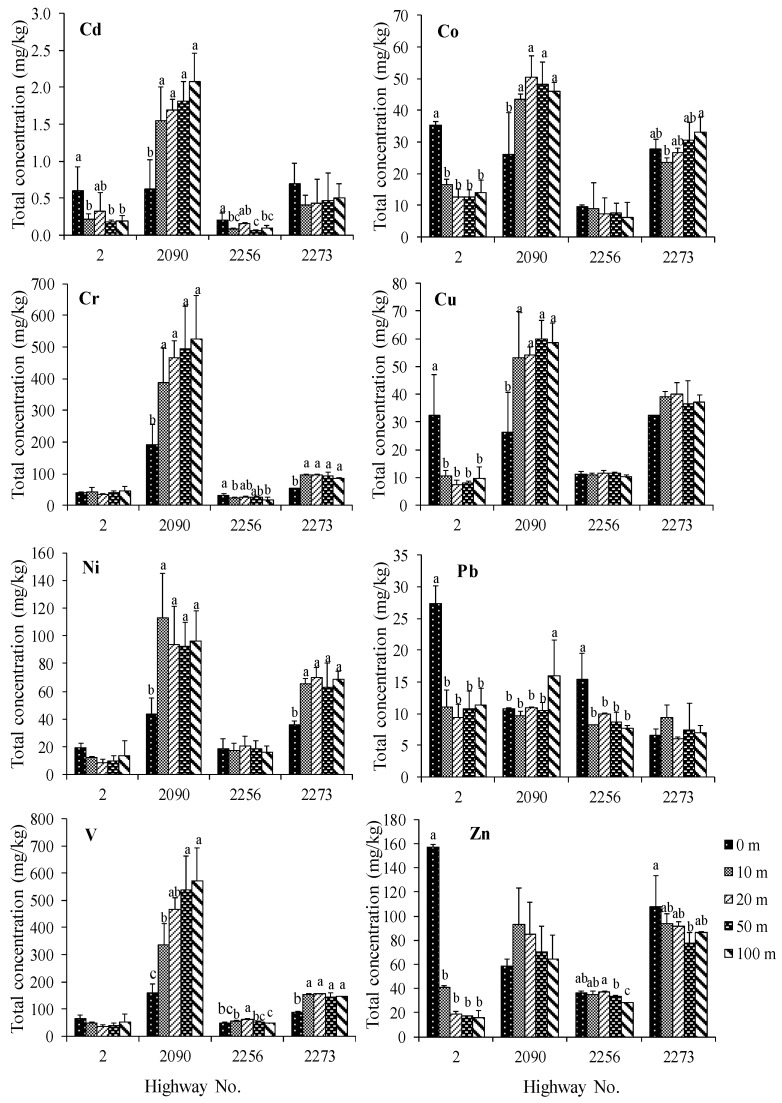
Mean total concentrations of Cd, Co, Cr, Cu, Ni, Pb, V, and Zn (mg kg^−1^) in maize growing soils at different distances from the road edge. The columns with different alphabets indicate significant differences and those without alphabets indicate insignificant differences in the soil concentrations (*p* < 0.05), and error bars indicate ±SD.

**Figure 4:**
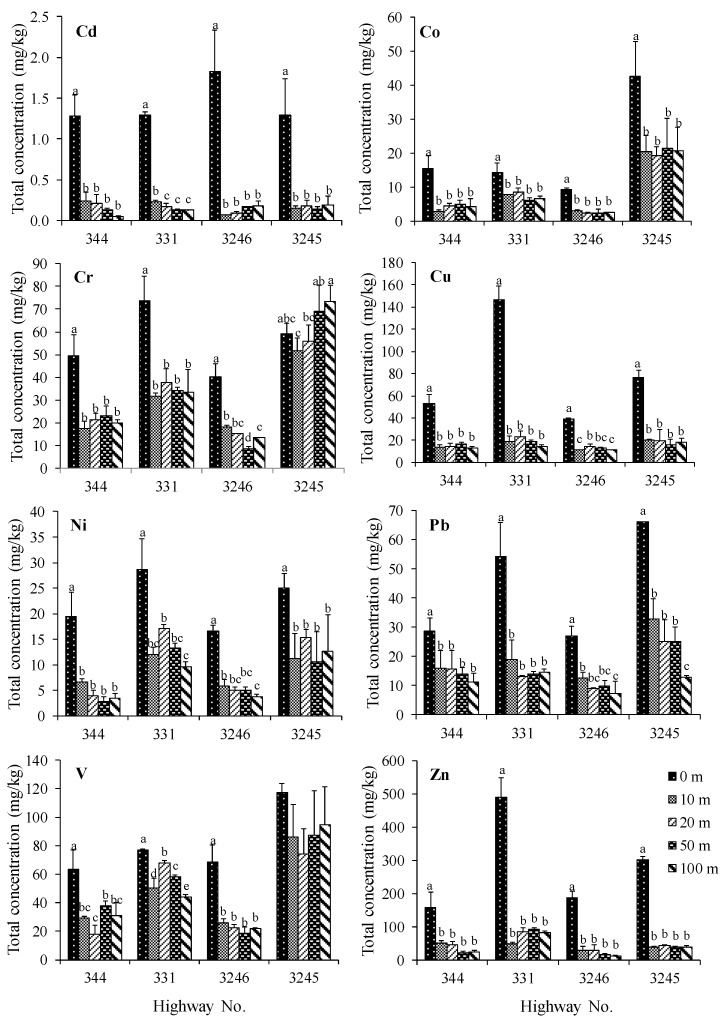
Mean total concentrations of Cd, Co, Cr, Cu, Ni, Pb, V, and Zn (mg kg^−1^) in sugarcane growing soils at different distances from the road edge. The columns with different alphabets indicate significant differences and those without alphabets indicate insignificant differences in the soil concentrations (*p* < 0.05), and error bars indicate ±SD.

The total concentrations of trace metals in the roadside agricultural soils (rice, maize, and sugarcane) are presented in Table 1. The concentrations of trace metals in rice growing soils were found in the following order: Zn > V > Cr > Cu > Pb ≈ Ni > Co > Cd. The roadside agricultural soils of highway 3032 contained the highest concentrations of most trace metals compared to the roadside soils of highways 340 and 3365. Importantly, the concentrations of Cd, Co, Cu, Pb, and Zn of most road edge soils were statistically significantly greater than the soils at a distance (10–100 m) from the road (*p* < 0.05), as shown in Figure 2. In roadside maize growing soils, the concentration of trace metals found were in the following sequence: V ≈ Zn > Cr > Cu ≈ Ni > Co > Pb > Cd. The concentration of most trace metals, except for Pb and Zn, in roadside maize growing soils, were found in following the order: highways no. 2090 > 2273 > 2 ≈ 2256, respectively. The Cd, Cu, Pb, and Zn concentrations found in most road edge soils from some highways were statistically significantly greater than the 10–100 m soils as in the roadside rice growing soils, as shown in Figure 3. The results from roadside sugarcane growing soils showed that most of the trace metals in most road edge soils were statistically significantly greater than the 10–100 m soils, as shown in Figure 4. The concentrations of trace metals in sugarcane growing soils were found in the following order: Zn > V > Cr ≈ Cu > Pb > Co > Ni > Cd. The concentration of some trace metals in the soils from highway 3245 were greater than the soils from the other highway soils.

In this study, the Co, Cr, Cu, Ni, V, and Zn concentrations in some roadside agricultural soils were found to be within, or higher than, the critical values for total trace metal concentrations in soils that may be toxic to plants [28]. Most of the trace metal concentrations in our study were higher than the mean trace metal concentration in Thai soils as reported by Zarcinas et al. [29]. Furthermore, trace metals in this study were higher than the concentrations of trace metals of roadside soils in Beijing [8] and the Tibetan Plateau, China [9], except for Cd content, which was lower than the roadside agricultural soils from Saraburi province, Thailand [30], as shown in Table 1.

### 3.3. Relationships of Soil Trace Metal Concentrations with Distance from the Highway and Traffic Density

#### 3.3.1. Distance from the Highway

The results showed the negative correlation of Cd, Cu, Pb, and Zn concentrations with increasing distance from the road edge (*r* = −0.176 *, −0.220 **, −0.323 **, and −0.359 **, respectively), as shown in Table 2, that is, the concentrations of Cd, Cu, Pb, and Zn in the roadside soil decreased with increasing distance from the road edge. This is presumably due to the deposition of atmospheric particulate matter enriched with Cd, Cu, Pb, and Zn into the roadside areas. Fuel combustion and the corrosion of vehicle parts, as well as battery and engine oil leaks, can substantially contribute to the elevation of trace metals in the atmospheric particulates [7,8,12,13]. Our results are similar to Maneerat et al. [31] who reported that Zn, Cd, and Pb accumulated in the total suspended particulate matter in some roadside areas of Thailand. Moreover, the road surface runoff water also contained Cd, Cu, Pb, and Zn, which can accelerate contamination of these metals in the roadside areas [32,33].

Furthermore, the results were consistent with the significantly elevated concentrations of Cd, Cu, Pb, and Zn concentrations in the road edge soils as compared to the 10–100 m soils, as shown in Figure 2, Figure 3 and Figure 4, which indicate the input of the traffic into the roadside soils [9,18,23]. Nevertheless, the differences in trace metal concentrations in soils at the distances of 10, 20, 50, and 100 m were unclear. This is possibly due to the influences of plantation activities such as plowing, irrigation, and plant uptake. This result is consistent with other studies reporting that traffic activities have an influence on trace metal contamination in roadside areas within 3–10 m of the road edge [21,22]. Moreover, the accumulation of certain trace metals in some roadside agricultural soils was also influenced by other trace metal sources such as soil parent materials, as well as fertilizer and pesticide applications [22,34].

#### 3.3.2. Traffic Density

Although several studies reported that trace metal distributions in roadsides soils were related to traffic density [8,18,23], the concentrations of Cd, Cu, Pb, and Zn in the studied roadside agricultural soils were not correlated with the traffic density, as shown in Table 2. This phenomenon could be due to the city development with extensive maintenance and expansion of roads, especially in high-traffic density highways. Furthermore, Co concentration in soils was negatively correlated with traffic density (*r* = −0.188 *).

### 3.4. Geochemical Affinity in Roadside Agricultural Soils

**Figure 5:**
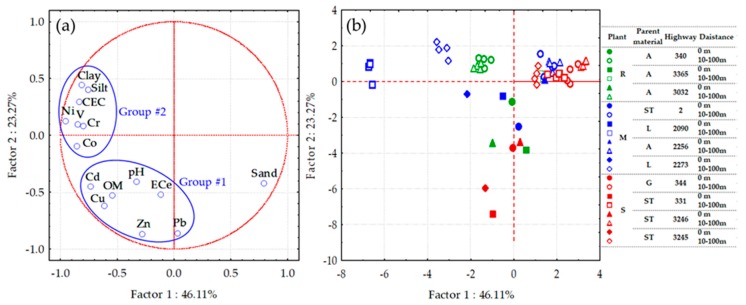
Principal component analysis (PCA) for metal concentrations and soil properties for bulk soil samples of roadside agricultural soils in Thailand: (**a**) distribution of metal contents and soil properties (variables); and (**b**) distribution of the studied soil samples in relation to plant types (R = rice; M = maize; S = sugarcane), soil parent materials (A = alluvial; ST = sandstone; L = limestone; and G = granite), highway numbers, and the distances from the road edge (cases).

The first two factors from the PCA accounted for 69.38% of the data variability, which signified the heterogeneous nature of the studied soils, as shown in Figure 5. Two element affinity groups were identified with some outliers (sand). These groups consisted of a Zn-affinity group #1 (Zn, Pb, Cu, Cd, OM, pH, and ECe), and a Ni-affinity group #2 (Ni, Co, V, Cr, Cd, clay, silt, and CEC). The first group, containing Zn, Pb, Cu, and Cd, was found to be enriched in the road edge soils (0 m distance). These trace metals were primarily related to street dust [4,6,7,31], particularly Pb, which was used as an additive to improve the octane rating of fuel [21]. Organic matter content was associated with this trace metal group, suggesting the high reactivity of organic matter for metal binding sites [35]. The high pH values of the road edge soils may indicate that these metals presenting in the soils could be found as metal–carbonate compounds.

The second group of elemental affinity was related to Ni, Co, V, and Cr, and their concentrations were elevated in the maize growing soils taken from highways 2090 and 2273 at the sampling distances of 10–100 m. These metal groups are typically substituted in the structure of natural clay minerals and iron oxides [36,37]. High concentrations of the Ni-affinity group in the soils could be interpreted as a consequence of their parent materials, which are derived mainly from limestone. Furthermore, plant types including rice, maize, and sugarcane were not correlated with the elemental affinities that the accumulations of trace metals exhibited in roadside agricultural soils in the present study.

### 3.5. Contamination Factor of the Trace Metals Concentration in Roadside Agricultural Soils

**Figure 6:**
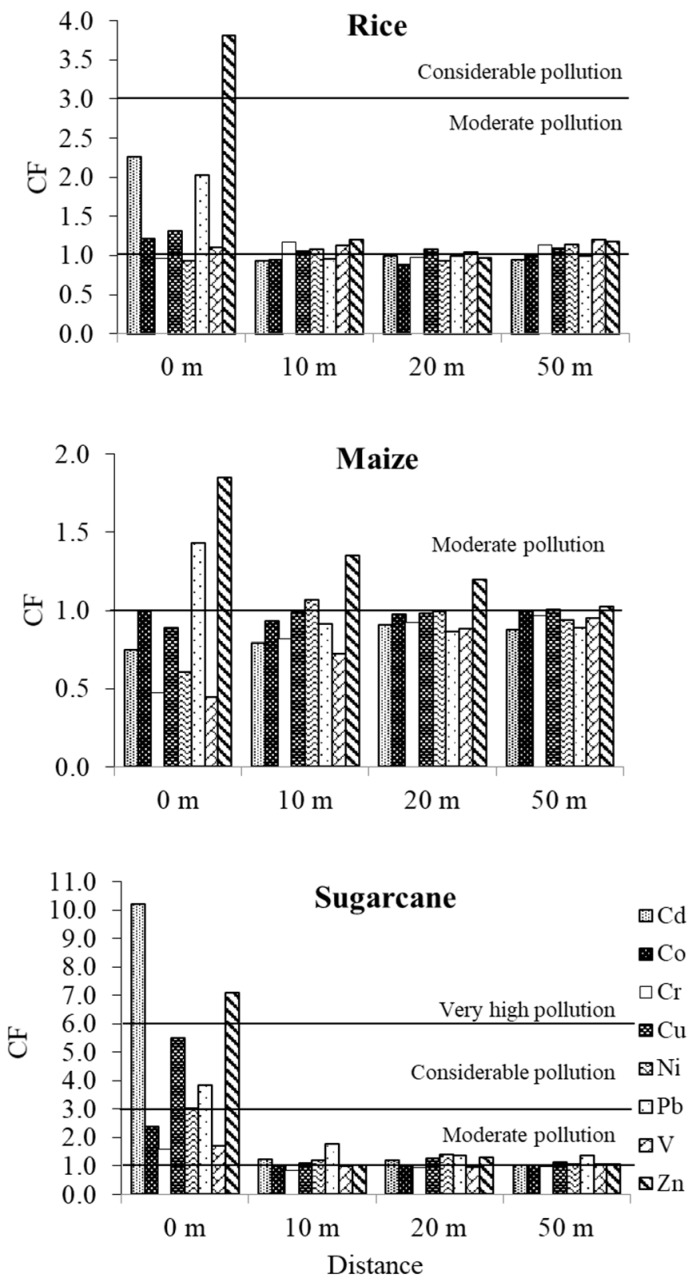
The contamination factor (CF) for Cd, Co, Cr, Cu, Ni, Pb, V and Zn in roadside agricultural soils under rice, maize and sugarcane cultivation.

Roadside agricultural soils under paddy rice showed CFs of trace metals (Cd, Co, Cr, Cu, Ni, Pb, V, and Zn) ranging from 0.9 to 3.8, indicating a range from no pollution (CF < 1) to considerable pollution (3 ≤ CF ≤ 6). However, in the road edge soils the Cd, Pb, and Zn levels (CF) were clearly higher than in the 10–50 m soils. The CFs of maize growing soils varied from 0.5 to 1.9, indicating no pollution to moderate pollution (1 ≤ CF < 3). For the sugarcane growing soils, the CFs (0.9–10.2) indicated a range from no pollution to very high pollution (CF > 6); the levels of trace metals at the road edge were clearly higher than those in the 10–50 m soils, as shown in Figure 6. The results showed that Zn, Cd, Pb, and Cu made a large contribution to pollution in the roadside soils, especially at the road edge. This suggests that the appropriate distance from the roadside for plantations should be more than 10 m away from the road edge so as to reduce the risk of soil contamination by Cd, Cu, Pb, and Zn.

## 4. Conclusions

The results showed that roadside agricultural soils contained Cd, Co, Cr, Cu, Ni, Pb, V, and Zn. The levels of trace metals in roadside agricultural soils for rice, maize, and sugarcane were as follows:

1. Rice: Zn > V > Cr > Cu > Pb ≈ Ni > Co > Cd;
2. Maize: V ≈ Zn > Cr > Cu ≈ Ni > Co > Pb > Cd;
3. Sugarcane: Zn > V > Cr ≈ Cu > Pb > Co > Ni > Cd.

The Cd, Cu, Pb, and Zn concentrations in most road edge soils were statistically significantly higher than those in the soils at a distance of 10–100 m (*p* < 0.05). Furthermore, Cd, Cu, Pb, and Zn showed a negative correlation with the distance from the highway, with the concentrations of Cd, Cu, Pb, and Zn decreasing with increasing distance from the road edge. However, the traffic density failed to make a clear contribution to the accumulation of trace metals in the roadside agricultural soils in this study, probably due to road expansion as a part of the urban development process. Moreover, mean concentrations of Cd, Cu, Pb, and Zn in road edge soils indicated that the soils were moderately to considerably polluted. This study proposes that the optimum spacing for plantations is more than 10 m away from the road edge to reduce the pollution risk of Cd, Cu, Pb, and Zn in roadside soils. Large accumulations of Cd, Cu, Pb, and Zn in roadside agricultural soils should be taken into consideration, as they have a high potential to enter into the food chain, which can cause adverse effects on biota and terrestrial environments.

## Figures and Tables

**Table 1:** Range values of total concentrations of trace metals in roadside agricultural soils at varying distances.

Plantation	Highway No.	Distance (m)	Trace Metals (mg kg^−1^)							
			Cd	Co	Cr	Cu	Ni	Pb	V	Zn
Rice	340	0–100	0.28–0.48	11.6–17.5	22.2–37.7	27.5–33.6	20.2–29.6	10.5–14.4	45.0–82.0	59.9–72.1
	3365	0–100	0.10–0.62	3.4–8.6	19.9–31.8	13.7–23.2	6.7–14.8	22.8–43.9	21.5–59.3	11.4–284
	3032	0–100	0.24–0.74	13.0–14.9	47.6–68.6	26.6–45.6	28.8–42.7	10.2–43.0	70.3–110	70.6–236
Maize	2	0–100	0.18–0.60	12.5–35.3	35.3–44.8	7.4–32.4	8.6–19.3	9.4–27.4	35.2–66.3	15.6–157
	2090	0–100	0.63–2.08	26.2–50.5	191–526	26.4–60.0	44.0–113	9.7–16.0	161–571	58.9–93.1
	2256	0–100	0.06–0.20	6.1–9.6	18.6–32.1	10.3–11.6	16.3–20.9	7.8–15.5	47.8–60.3	28.3–37.3
	2273	0–100	0.41–0.69	23.5–33.0	54.0–95.8	32.4–39.9	36.0–70.2	6.1–9.4	86.6–157	77.9–108
Sugarcane	344	0–100	0.05–1.29	2.8–15.4	17.5–49.7	13.0–53.2	2.83–19.43	11.1–28.8	17.8–63.2	22.5–159
	331	0–100	0.13–1.29	6.1–14.3	31.6–73.8	14.6–146	12.1–28.7	13.1–54.3	44.0–77.8	48.5–489
	3246	0–100	0.07–1.83	2.4–9.2	8.8–40.4	11.2–39.0	3.8–16.7	7.2–27.1	18.3–68.6	13.2–188
	3245	0–100	0.14–1.30	19.2–42.6	51.7–73.3	15.6–76.2	10.6–25.1	12.9–66.1	74.4–117	38.5–302
Critical value [28]			3–8	25–50	75–100	60–125	100	100–400	5–10	50–100
Thailand [29]			0.03	6.0	25.2	14.1	13.5	17.5	-	23.9
Saraburi, Thailand [30]			1.3–3.7	-	-	-	-	16.1–30.6	-	68.3–151.5
Beijing, China [8]			0.22	-	61.9	29.7	26.7	35.4	-	-
Tibetan Plateau, China [9]			0.13	8.1	54.3	18.4	20.4	18.5	–	51.7

**Table 2:** Pearson correlation between the distance from the road, traffic density and total concentrations of trace metals in roadside agricultural soils (*n* = 165).

Trace Metals	Distance	Traffic Density
Total Cd	−0.176 *	−0.108
Total Co	−0.063	−0.188 *
Total Cr	0.061	−0.125
Total Cu	−0.220 **	−0.109
Total Ni	0.001	−0.048
Total Pb	−0.323 **	−0.129
Total V	0.075	−0.104
Total Zn	−0.359 **	−0.104

* Correlation is significant at the 0.05 level (2-tailed) ** Correlation is significant at the 0.01 (level 2-tailed).

## Supplementary Materials

The following are available online at https://www.mdpi.com/1660-4601/16/5/714/s1, Table S1: Details for sampling locations with the crop type, highway number, and traffic density for each highway. Table S2. Some physical and chemical properties of the roadside agricultural soils.
