# Supplementary material for: The Distribution of Trace Metals in Roadside Agricultural Soils, Thailand
Source: Int J Environ Res Public Health. 2019 Feb 27;16(5):714. doi: 10.3390/ijerph16050714 (PMC6427218; doi:10.3390/ijerph16050714)
Supplement: Supplementary file 1 [file ijerph-16-00714-s001.pdf]

International Journal of
Environmental Research and Public Health

MDPI

Article

The Distribution of Trace Metals in Roadside Agricultural Soils, Thailand

Nattanan Krailertrattanachai, Daojarus Ketrot * and Worachart Wisawapipat

Supplementary Materials

Table S1. Details for sampling locations with the crop type, highway number, and traffic density for each highway.

Plantation	Highway no.	Location (UTM 47P)		Traffic Density (million vehicles/kilometer/year)
		mE	mN	
**Rice**	**340**	0624429	1614325	> 100
		0616548	1651133	
		0618340	1630772	
	3365	0606499	1632946	60–90
		0610454	1632313	
		0614745	1631388	
	3032	0621783	1636484	< 30
		0632060	1639349	
		0626065	1636248	
Maize	2	0817071	1651480	> 200
		0820546	1653039	
		0808746	1647736	
	2090	0758560	1612782	> 100
		0759310	1610855	
		0757981	1615506	
	2256	0772016	1673638	60–90
		0775704	1674180	
		0785149	1676190	
	2273	0737987	1641610	30 – 60
		0737821	1626319	
		0738832	1634692	
Sugarcane	344	0742882	1461825	> 200
		0753188	1454340	
		0748717	1455926	
	331	0743377	1466292	100 – 200
		0743442	1466590	
		0745927	1475996	
	3246	0743588	1482654	60 – 90
		0743356	1483153	
		0741064	1484919	
	3245	0764217	1465575	30 – 60
		0764261	1464957	
		0764362	1469976	

Table S2. Some physical and chemical properties of the roadside agricultural soils.

Plantation	Highway no.	Soil parent material	Distance (m)	Soil texture	Sand	Silt	Clay	pH 1:1	ECe	OM	CEC
					(-----------g kg⁻¹---------)			(H_2O)	(dS m^{-1})	(g kg^{-1})	($cmol_c$ kg^{-1})
**Rice**	**340**	**Alluvium**	0	**Sandy Clay Loam**	**495**	**229**	**276**	**7.09**	2.4	19	13.3
			10-100	Silty Clay	181	409	410	6.70	0.8	26	18.0
	3365	Alluvium	0	Sandy Loam	757	131	112	7.44	1.0	46	12.1
			10-100	Sandy Clay Loam	598	180	222	6.59	0.6	24	15.8
	3032	Alluvium	0	Sandy Loam	593	219	188	7.15	1.0	54	16.8
			10-100	Loam	250	394	356	6.60	0.8	36	23.5
Maize	2	Sandstone	0	Sandy Loam	777	95	128	7.77	1.0	29	9.2
			10-100	Sandy Loam	682	143	175	6.73	0.4	12	12.1
	2090	Limestone	0	Sandy Loam	737	131	132	7.93	0.7	28	9.8
			10-100	Clay	224	347	429	6.58	0.5	30	25.6
	2256	Alluvium	0	Sandy Loam	695	143	162	7.29	0.5	21	10.5
			10-100	Sandy Loam	670	123	177	6.61	0.5	11	11.4
	2273	Limestone	0	Clay Loam	449	274	277	7.63	0.8	61	23.9
			10-100	Silty Clay	136	457	407	7.11	0.4	20	28.0
Sugarcane	344	Granite	0	Sandy Loam	775	114	111	7.45	1.6	41	8.9
			10-100	Sandy Loam	769	124	107	5.89	0.6	12	6.7
	331	Sandstone	0	Loamy Sand	852	58	90	7.40	0.9	30	6.9
			10-100	Sandy Loam	745	93	162	6.35	0.3	11	10.0
	3246	Sandstone	0	Loamy Sand	825	70	105	7.15	1.0	37	9.8
			10-100	Loamy Sand	816	85	99	5.69	0.2	7	3.5
	3245	Sandstone	0	Loamy Sand	808	104	88	6.81	1.0	47	11.8
			10-100	Sandy Loam	657	173	170	5.94	0.7	13	15.1
